# The Impact of the COVID-19 Pandemic on Health Behaviours of Pregnant Women in Poland: A Cross-Sectional Study

**DOI:** 10.3390/nu16010088

**Published:** 2023-12-27

**Authors:** Kinga Janik, Grazyna Iwanowicz-Palus, Mateusz Cybulski

**Affiliations:** 1 Department of Integrated Medical Care, Faculty of Health Sciences, Medical University of Bialystok, M. Sklodowskiej-Curie 7A Street, 15-096 Bialystok, Poland; mateusz.cybulski@umb.edu.pl; 2 Department of Development in Midwifery, Faculty of Health Sciences, Medical University of Lublin, Staszica 4/6 Street, 20-081 Lublin, Poland; spupalus@umlub.pl

**Keywords:** COVID-19, Health Behaviour Inventory, pregnant women, lifestyle, health behaviours

## Abstract

Health behaviours of pregnant women should promote an optimal course of pregnancy and maternal health. The purpose of this study was to assess the impact of COVID-19 pandemic on pregnant women’s health behaviours (proper eating habits with a particular focus on the type of food consumed; preventive behaviours in terms of compliance with health recommendations and obtaining information on health and disease; healthy practices—daily habits in terms of sleep, rest and physical activity; as well as positive mental attitudes—avoiding strong emotions, stress, and depressing situations, determined by the frequency of each behaviour reported by the respondents). The study included women at different stages of pregnancy and women who were pregnant during the COVID-19 pandemic. A total of 355 women participated in the study. The study used a proprietary questionnaire and the Health Behaviour Inventory (HBI). The overall health behaviour score during the pandemic was higher (85.87) than the pre-pandemic score (82.16). There was a statistically significant difference between the total pre-pandemic and during-pandemic HBI scores. Additionally, there was an increase in the total score in each of the health behaviour domains during the pandemic period compared to the pre-pandemic results. During the COVID-19 pandemic, pregnant women presented a statistically significantly higher rate of health behaviours, as measured with the HBI, indicating that respondents were more likely to engage in health-promoting behaviours. The study has shown a positive change in pregnant women’s eating habits, which can potentially affect the health of the population in the future.

## 1. Introduction

Health behaviours of childbearing women should promote an optimal course of pregnancy and maternal health. Therefore, pregnant women should follow basic recommendations for a health-promoting lifestyle, especially when it comes to the type of diet and balanced physical activity [1]. Lifestyle plays a major role in maintaining good health potential and determines the quality of life of women during pregnancy. However, pregnant women tend to discontinue many activities, replacing them with passive ones [1].

Inappropriate health behaviours in pregnancy are one of two causes of postnatal complications and deaths from pregnancy complications in the United States [2,3,4]. Health risks for pregnant women are mainly determined by sociodemographic factors, such as low socioeconomic status and poor education [5]. On the other hand, the sexual health and health behaviours of pregnant women are equally important factors, as they have an impact on both maternal and foetal well-being. However, the lack of awareness in this regard or failure to make changes despite having proper knowledge raises concerns. Women should change or abandon risky behaviours and develop or maintain health-promoting ones that include a healthy diet, physical activity and abstinence from stimulants such as alcohol, cigarettes and excessive coffee consumption. Furthermore, pregnant women are recommended to use dietary supplements such as folic acid and vitamins, keep regular sleeping hours and undergo screening [6]. On the positive side, a significant number of women modify their lifestyles, reducing smoking, caffeine and alcohol consumption immediately before and during pregnancy and increasing their intake of fruits and vegetables during pregnancy [7,8]. Nevertheless, many women undertake unhealthy practices regardless of the existing evidence and healthcare system recommendations that promote a healthy lifestyle.

The COVID-19 pandemic contributed to Poland’s worst post-World War II health crisis. High levels of perceived stress, such as that caused by social distance and isolation, contributed to poorer diet, lack of physical inactivity, as well as shorter and poorer sleep [9]. Pregnant women were more likely to experience adverse lifestyle changes in response to the COVID-19 pandemic due to the accumulation of multiple sources of perceived stress [10,11]. In particular, the pandemic had a negative impact on the mental health of pregnant and postpartum women, who experienced increased anxiety, depression, relationship tension and social isolation [12]. According to a study conducted during the COVID-19 pandemic, anxiety affected 18% to 56% of perinatal and postpartum women, with anxiety and depressive disorders present in 10–33% of women [13]. Pregnant women have also been shown to have higher rates of anxiety due to fear of intrauterine transmission of the SARS-CoV-2 virus and its possible consequences on their unborn child [14]. Additionally, given the numerous restrictions on hospital wards during the pandemic, including bans on visits or the presence of family members at birth, the COVID-19 pandemic clearly had a significant impact on the lives of pregnant and postpartum women [14].

Many authors have evaluated and described the impact of the COVID-19 pandemic on the mental health of pregnant women. However, there is insufficient data on the impact of the pandemic on pregnant women’s lifestyles and health behaviours, which play an important role among women of reproductive age. Therefore, the purpose of this study was to assess the impact of the COVID-19 pandemic on pregnant women’s health behaviours.

## 2. Materials and Methods

### 2.1. Participants

The study included women at various stages of pregnancy and women who were pregnant during the COVID-19 pandemic. A total of 355 women participated in the study. The age of respondents ranged from 18 to 47 years. Most of them were married (79%). Almost 62% of respondents had a university education. Less than 32% of respondents declared a rural place of residence, while the rest of the women resided in Polish cities. More than 64% of respondents described their socioeconomic status as “good”. Nearly one-third of respondents were expecting their first child, while the remaining women had between one and five children. Nearly 12% of women declared having COVID-19 during pregnancy. Detailed sociodemographic characteristics are shown in Table 1.

### 2.2. Study Design and Data Collection

The study was conducted between 2 January 2023 and 30 June 2023 in a voluntary group. A link to a dedicated questionnaire on the Webankieta platform was posted on social networks in appropriately profiled discussion groups.

Data from before the pandemic were collected as part of the same questionnaire, and the Health Behaviour Inventory was completed twice—the first time referring to health behaviours 1 month before the outbreak of the pandemic and the second time verifying currently undertaken health behaviours.

The study was anonymous. Participation in the study was voluntary and tantamount to consent to the processing of the data obtained for research purposes. Ongoing pregnancy or pregnancy during the COVID-19 pandemic was an inclusion criterion. Each participant could withdraw from the study at any time.

The responses of respondents were recorded on the platform, then downloaded as raw data and analysed statistically using a dedicated programme.

The platform recorded 592 displays. The level of full completion of the questionnaire was 60% in relation to the number of displays, with 28 unfinished questionnaires.

### 2.3. Measures

The study used a proprietary questionnaire designed for the purpose of this study. The questionnaire contained questions on sociodemographic characteristics and seven closed-ended, single-choice questions, including one in which an affirmative response required one’s own answer to be entered. The questions in the questionnaire concerned contracting and vaccinating against COVID-19 and accessibility to physicians and midwife care during the COVID-19 pandemic. The respondents were also asked about their anxiety and fear related to the pandemic.

Furthermore, the study used the Health Behaviour Inventory (HBI) by Z. Juczynski [15]. HBI is a tool consisting of 24 statements that describe various types of health-related behaviours. The tool measures the overall level of health behaviours and their four dimensions: proper eating habits with a particular focus on the type of food consumed (statements: “I eat a lot of vegetables and fruits”, “I limit the consumption of products such as animal fats and sugar”, “I care about proper nutrition”, “I avoid eating food with preservatives”, “I avoid salt and strongly salted foods”, “I eat whole grain bakery products”); preventive behaviours in terms of compliance with health recommendations and obtaining information on health and disease (statements: “I avoid colds”, “I have written down the telephone numbers of the emergency services”, “I follow medical recommendations resulting from my medical tests”, “I regularly undergo medical examinations”, “I try to find out how others avoid illness”, “I strive to obtain medical information and understand the causes of health and disease”); healthy practices—daily habits in terms of sleep, rest and physical activity (statements: “I get enough rest”, “I avoid overwork”, “I control my weight”, “I sleep enough”, “I limit smoking”, “I avoid excessive physical exercise”); as well as positive mental attitudes—avoiding strong emotions, stress, and depressing situations (statements: “I take seriously the advice of people expressing concern about my health”, “I avoid situations that have a depressing effect on me”, “I try to avoid too strong emotions, stress and tensions”, “I have friends and a settled family life”, “I avoid feelings such as anger, anxiety and depression”, “I think positive”), determined by the frequency of each behaviour reported by the respondents [15].

The respondents indicated how often they were involved in the given health-related activities by rating each of the behaviours listed in the Inventory on a five-point scale, where: 1—almost never, 2—rarely, 3—occasionally, 4—often, 5—almost always. The overall HBI score ranged from 24–120. The higher the score, the higher the level of declared health behaviour. The level of health behaviours in the four dimensions was calculated separately as the mean score in each category, i.e., the total score divided by 6. Cronbach’s alpha for the HBI is 0.85, and for its four subscales, it ranges from 0.60 to 0.65 [15].

### 2.4. Procedure and Ethical Considerations

The study was conducted in accordance with the recommendations and was reviewed and approved by the Bioethics Committee of the Medical University of Bialystok (resolution no. APK.002.432.2022). All participants gave written informed consent in accordance with the Declaration of Helsinki.

### 2.5. Statistical Analysis

Statistica 13.3 PL software (StatSoft Poland, Krakow, Poland) was used for statistical analysis. The variables analysed were dichotomous, interval or ordinal. Basic descriptive statistics were used for those variables. The chi-square test was used to assess the existence of relationships between qualitative variables. The normality of the distribution of variables, including differences, was assessed using the Shapiro–Wilk test. No normality of distribution was found; they were analysed using non-parametric tests. The significance of the differences between the two groups was verified with the Mann–Whitney U-test and for dependent variables—the Wilcoxon matched-pairs test. The results were statistically significant at *p* < 0.05.

## 3. Results

Table 2 shows an analysis of questions from the authors’ questionnaire. Slightly more than 52% of women surveyed declared that they had been vaccinated against COVID-19. Less than 12% of respondents answered affirmatively to the question “Have you contracted COVID-19 during pregnancy?”. The fear of contracting COVID-19 during pregnancy was reported by ¼ of the women surveyed. Problems with access to a gynaecologist during the pandemic period were reported by 20% of pregnant women, while a much smaller number of respondents (8%) declared problems with access to a midwife. More than half of the respondents (54%) confirmed experiencing anxiety or fear during the COVID-19 pandemic. A total of 17 women (5%) declared experiencing mental disorders, including depression in 10, anxiety in 5, social phobia in 1 and neurosis in 1 respondent.

Table 3 shows the overall health behaviour rates according to HBI from two measurements—before and during the COVID-19 pandemic. The aggregated results are for the entire group of respondents. It was observed that the score measured during the pandemic was higher (85.87) than the pre-pandemic score (82.16), indicating an overall increase in health behaviours during the pandemic among pregnant respondents. The obtained results are within the average range. Additionally, there was a statistically significant difference between the total pre- and during-pandemic HBI scores, i.e., an obvious increase in HBI scores was observed during the pandemic period. This table shows the HBI scores by health behaviour category, i.e., proper eating habits, preventive behaviours, health practices, and positive mental attitudes. The analysis has shown that there was a statistically significant increase in the sum in each of the health behaviour domains during the pandemic compared to the results for the pre-pandemic COVID-19 period.

Table 4 shows detailed HBI scores from two measurements—before and during the COVID-19 pandemic. HBI scores are reported by sociodemographic data. It was shown that the analysed variables (place of residence, number of children, trimester of pregnancy, vaccination against COVID-19, anxiety and fear about the pandemic) had a statistically significant impact on the improvement of health behaviours undertaken before and during the pandemic. It was noted that the HBI score was higher during the pandemic in each group of female respondents. Rural residents had a slightly higher score than urban residents. Primiparous (85.30) and multiparous (86.15) women had similar scores during the pandemic. As the trimester of pregnancy increased, HBI scores were slightly lower, with 87.70, 87.20, and 85.11 in the first, second, and third trimesters of pregnancy, respectively. The study found that vaccinating pregnant women against COVID-19 had no effect on HBI scores. Perceived anxiety and fear due to the pandemic were not reflected in the HBI scores. Pregnant women who experienced anxiety scored 85.68 vs. 86.09 for those who did not, indicating little difference between the groups.

It was shown that the analysed variables (place of residence, number of children, vaccination against COVID-19, anxiety and fear about the pandemic) did not have a statistically significant impact on the improvement of proper eating habits, positive mental attitudes and preventive behaviours undertaken before and during the pandemic. Only vaccinated and unvaccinated people against COVID-19 differed significantly in terms of preventive behaviours undertaken during the COVID-19 pandemic (*p* = 0.021). Detailed data is presented in Table 5, Table 6, Table 7 and Table 8.

## 4. Discussion

The study presented here is among the few to assess the impact of the COVID-19 pandemic on pregnant women’s health behaviours. The study found that pregnant women had higher HBI scores during the pandemic period than before the pandemic. This indicates an increase in positive health behaviours during the COVID-19 pandemic. Proper health habits, preventive behaviours and health practices increased in the pregnant women surveyed. The smallest increase can be seen in the category of positive mental attitudes.

In addition to the obvious negative effects of the COVID-19 pandemic, there are also reports of positively experienced changes in attitudes and behaviours, which were described in their research by Büssing et al. [16]. Immediately after the first lockdown, which lasted about 3 months, people were more aware of their relationships, intensified and valued them more than before, went for walks in the fresh air more often, perceived nature more intensely, consciously devoted more time to silence, and enjoyed peaceful moments of reflection, they were more attentive to what they considered truly important in life and used this extra time to reflect on the meaning and purpose of life [16]. O’Brien et al. showed that this mindful approach to difficult life situations has a protective effect on health behaviours [17].

Jarraya et al. [18], in a study conducted in 2021–2022 in a group of unvaccinated women who were hospitalised for childbirth and who gave birth during infection, observed more asymptomatic and minor forms that influenced the method of delivery and indications for caesarean delivery and reduced postnatal morbidity and mortality. The same study found that unvaccinated women giving birth may require oxygen support and sometimes admission to an intensive care unit. This highlights the importance of vaccination. In our study, only just over half of pregnant women (52%) were vaccinated against COVID-19.

Dietary habits that can contribute to overall positive health include: (1) increased consumption of whole grains, which is associated with a reduced risk of coronary heart disease, stroke, other cardiovascular diseases and cancers, as well as a reduced risk of mortality from cardiovascular diseases, cancers, respiratory diseases, diabetes and infectious diseases [19,20,21]; (2) consumption of fresh fruits and vegetables, which provide energy and dietary fibre, which promotes the feeling of satiety and has a positive effect on the functioning of the digestive tract, cholesterol levels and glycaemic control, are also a valuable source of polyphenols, phytosterols and carotenoids [22], and their consumption inversely correlates with risk of non-communicable diseases, including hypertension [23], cardiovascular diseases [24,25], chronic obstructive pulmonary disease [26], lung cancer [27] and metabolic syndrome [28]; (3) consumption of unsaturated fats, which are associated with a reduced risk of cardiovascular diseases and mortality for this reason [29,30]; (4) supplementation or consumption of foods rich in omega-3 fatty acids, particularly eicosapentaenoic acid (EPA) and docosahexaenoic acid (DHA) (e.g., fatty fish), which provide cardioprotection, prevention of cognitive decline, reduction of inflammation and maintaining muscle mass and improving systemic insulin resistance [31,32,33] and (5) adequate water intake, which not only provides hydration, but also carries micronutrients, including trace elements and electrolytes, and can also provide as much as 20% of the daily recommended intake of calcium and magnesium [34]. On the other hand, millions of people lead unhealthy lifestyles, which include malnutrition, unhealthy diet, smoking, alcohol consumption, low physical activity, etc. and which take over the dominant form of lifestyle in society. An unhealthy lifestyle determines the occurrence of metabolic diseases, problems with joints and skeleton, cardiovascular diseases, hypertension, overweight, etc. [35]. Therefore, special attention should be paid to the relationship between lifestyle and health, especially among pregnant women.

Boguszewski et al. [1] conducted a study on pregnant women from Warsaw, using the HBI to assess healthy behaviours. Their mean scores were 87.56, similar to the mean scores obtained in our study, where pregnant women scored 82.16 before the pandemic and 85.87 during the COVID-19 pandemic. Luong et al. [36] investigated the impact of healthy eating habits and health competencies on anxiety and depression in pregnant women during the COVID-19 pandemic. The study found that pregnant women with high levels of healthy eating behaviours and health competencies were less likely to experience anxiety and depression [36]. The positive correlation between a healthy diet and better mental health has also been confirmed by other researchers [37,38]. The COVID-19 pandemic crisis limited access to healthy food [39]. Therefore, healthy eating habits should be promoted among pregnant women to improve their mental health and consequently improve the overall health of the mother and her infant. A detailed analysis of HBI scores in our study confirmed that positive mental attitudes did not change significantly among pregnant women.

More than half of the pregnant women included in the study answered affirmatively to the question about experiencing anxiety and fear during the pandemic. An American study found moderate to severe anxiety in 42% of pregnant women [40]. An Irish study showed that up to 50.7% of pregnant women had been concerned about their health since the beginning of the COVID-19 pandemic [41]. Our earlier studies also demonstrated that pregnant women showed anxiety of SARS-CoV-2 infection, but this oscillated at a rather moderate level [42,43].

A study conducted among pregnant women in China assessed food intake and diet quality during the COVID-19 pandemic, and the authors showed that the overall diet quality of the respondents was moderately unbalanced [44]. Another study conducted in China showed moderate to severe dietary imbalance [45]. Kebbe et al. [46] showed that women also maintained or changed their eating behaviours during pregnancy. Most women (77%) reported increased daily vitamin intake during pregnancy. Most also reported snacking more frequently (72%), especially before bedtime (40.5%); eating more (60.9%), but eating similarly at night (63.9%) and not counting calories as often as before pregnancy (50.8%). However, they reported maintaining the amount of fat (64%), salty foods (56.5%), and fried and fast food (47.1%) during pregnancy. Although in our study the overall dietary balance was not analysed, a conclusion may be ventured based on the results presented that the pregnant women surveyed improved their eating habits to a greater or lesser extent during the COVID-19 pandemic.

### Limitations

This study has some limitations. The results presented here were obtained in a study based on a subjective assessment of the feelings experienced by pregnant women. Furthermore, the data obtained in the study concern only pregnant volunteers and may differ significantly from data from pregnant women who are not volunteers. The study used a standardised scale, which is a sensitive research tool, but its evaluation relies on subjective feelings and does not include objective criteria for clinical symptoms, which may contribute to false-positive results. Additionally, since the Health Behaviour Inventory is a Polish tool, global data on pregnant women using this score are missing. Moreover, there is a risk of response bias because the study was conducted in 2023 and also addressed health behaviours undertaken before the COVID-19 pandemic. Another limitation was the number of women participating in the study. The small group included in the study does not allow for generalising the results to the entire population of pregnant women in Poland. However, despite the limitations, the results presented here can serve as a reference for further studies assessing pregnant women’s health behaviours during the COVID-19 pandemic both in Poland and worldwide.

## 5. Conclusions

During the COVID-19 pandemic, pregnant women had a statistically significantly higher rate of health behaviours as measured by HBI, indicating that respondents were more likely to engage in health-promoting behaviours. The study confirmed a positive change in pregnant women’s eating habits, which will potentially affect the health of the population in the future.

## Figures and Tables

**Table 1:** Sociodemographic characteristics of respondents.

Sociodemographic Variable		Study Group	
		*n*	%
Education	elementary	7	2
	middle school	8	2
	basic vocational	16	4
	secondary	105	30
	higher	219	62
Marital status	married	280	79
	divorced	15	4,5
	single	44	12
	in a non-marital relationship	16	4,5
Place of residence	urban	239	67
	rural	116	33
Socioeconomic status	excellent	63	18
	good	229	64.5
	average	62	17.5
	poor	1	0
Number of children	pregnant with first child	116	33
	one	138	39
	two	66	19
	three	29	8
	four	4	1
	five	2	0

**Table 2:** Characteristics of the study group in terms of COVID-19, vaccinations against COVID-19 and the mental state of the respondents.

	Yes		No	
	*n*	%	*n*	%
Are you concerned about contracting COVID-19 during your pregnancy?	90	25	162	46
Did you have COVID-19 during your pregnancy?	42	12	313	88
Are you vaccinated against COVID-19?	186	52	169	48
Did you have any problems with access to medical care while pregnant during the pandemic?	71	20	284	80
Did you have any problems with access to midwifery care while pregnant during the pandemic?	30	8	325	92
Did you experience anxiety or fear during the pandemic?	191	54	164	46
Did you experience any mental disorders during the COVID-19 pandemic?	17	5	338	95

**Table 3:** Pre- and during-pandemic overall HBI scores and HBI scores for different health behaviour domains among pregnant women before and during the COVID-19 pandemic.

		*n*	M	Min	Max	SD	*p*
Overall health behaviour rate	before pandemic	355	82.16	41	115	12.91	<0.001 *
	during pandemic		85.87	51	120	13.03	
Proper eating habits	before pandemic	355	3.38	1.67	5	0.65	<0.001 *
	during pandemic		3.51	1.83	5	0.66	
Preventive behaviours	before pandemic	355	3.36	1.33	5	0.69	<0.001 *
	during pandemic		3.60	1.5	5	0.66	
Positive mental attitudes	before pandemic	355	3.61	1.67	5	0.67	<0.001 *
	during pandemic		3.70	1.5	5	0.69	
Health practices	before pandemic	355	3.34	1.67	5	0.63	<0.001 *
	during pandemic		3.50	1.5	5	0.69	

Abbreviations: M—arithmetic mean, Min—minimum, Max—Maximum, SD—standard deviation, *p*—probability value (Wilcoxon matched-pairs test), *—statistically significant.

**Table 4:** Comparison of HBI scores before and during the COVID-19 pandemic in selected groups.

Sociodemographic Data		*n*	M ± SD Pre-Pandemic HBI Score	M ± SD during-Pandemic HBI Score	*p*
place of residence	urban	239	82.21 ± 13.09	85.38 ± 13.75	<0.001 *
	rural	116	82.07 ± 12.59	86.88 ± 11.40	<0.001 *
number of children	primipara	116	81.90 ± 13.44	85.30 ± 14.98	<0.001 *
	multipara	239	82.30 ± 12.68	86.15 ± 11.99	<0.001 *
trimester	I	33	83.18 ± 14.10	87.70 ± 15.44	<0.001 *
	II	59	82.33 ± 14.83	87.20 ± 13.22	<0.001 *
	III	206	82.54 ± 12.57	85.11 ± 13.20	<0.001 *
COVID-19 vaccination	yes	186	82.18 ± 13.41	86.08 ± 13.19	<0.001 *
	no	169	82.15 ± 12.39	85.64 ± 12.89	<0.001*
anxiety and fear about the pandemic	yes	191	81.84 ± 12.91	85.68 ± 12.59	<0.001 *
	no	164	82.54 ± 12.95	86.09 ± 13.57	<0.001 *

Abbreviations: M—arithmetic mean, SD—standard deviation, *p*—probability value (Wilcoxon matched-pairs test), *—statistically significant.

**Table 5:** Comparison of proper eating habits before and during the COVID-19 pandemic in selected groups.

Sociodemographic Data		*n*	M ± SD Pre-Pandemic Proper Eating Habits	*p*	M ± SD during-Pandemic Proper Eating Habits	*p*
place of residence	urban	239	3.38 ± 0.68	0.780	3.49 ± 0.67	0.606
	rural	116	3.38 ± 0.59		3.55 ± 0.63	
number of children	primipara	116	3.31 ± 0.67	0.076	3.44 ± 0.74	0.073
	multipara	239	3.41 ± 0.63		3.54 ± 0.61	
COVID-19 vaccination	yes	186	3.38 ± 0.66	0.746	3.51 ± 0.63	0.683
	no	169	3.38 ± 0.63		3.51 ± 0.68	
anxiety and fear about the pandemic	yes	191	3.38 ± 0.66	0.704	3.49 ± 0.65	0.872
	no	164	3.38 ± 0.64		3.52 ± 0.67	

Abbreviations: M—arithmetic mean, SD—standard deviation, *p*—probability value (Mann–Whitney U-test).

**Table 6:** Comparison of preventive behaviours before and during the COVID-19 pandemic in selected groups.

Sociodemographic Data		*n*	M ± SD Pre-Pandemic Preventive Behaviours	*p*	M ± SD during-Pandemic Preventive Behaviours	*p*
place of residence	urban	239	3.36 ± 0.69	0.802	3.58 ± 0.69	0.436
	rural	116	3.36 ± 0.70		3.64 ± 0.59	
number of children	primipara	116	3.36 ± 0.70	0.949	3.58 ± 0.74	0.947
	multipara	239	3.36 ± 0.69		3.61 ± 0.62	
COVID-19 vaccination	yes	186	3.40 ± 0.68	0.131	3.66 ± 0.66	0.021 *
	no	169	3.31 ± 0.70		3.53 ± 0.67	
anxiety and fear about the pandemic	yes	191	3.40 ± 0.72	0.200	3.65 ± 0.66	0.080
	no	164	3.31 ± 0.66		3.54 ± 0.66	

Abbreviations: M—arithmetic mean, SD—standard deviation, *p*—probability value (Mann–Whitney U-test), *—statistically significant.

**Table 7:** Comparison of positive mental attitudes before and during the COVID-19 pandemic in selected groups.

Sociodemographic Data		*n*	M ± SD Pre-Pandemic Positive Mental Attitudes	*p*	M ± SD during-Pandemic Positive Mental Attitudes	*p*
place of residence	urban	239	3.63 ± 0.67	0.616	3.69 ± 0.73	0.815
	rural	116	3.58 ± 0.67		3.73 ± 0.61	
number of children	primipara	116	3.56 ± 0.73	0.307	3.62 ± 0.78	0.107
	multipara	239	3.64 ± 0.64		3.75 ± 0.64	
COVID-19 vaccination	yes	186	3.60 ± 0.69	0.916	3.71 ± 0.71	0.747
	no	169	3.63 ± 0.65		3.70 ± 0.67	
anxiety and fear about the pandemic	yes	191	3.58 ± 0.66	0.299	3.66 ± 0.68	0.247
	no	164	3.66 ± 0.68		3.75 ± 0.71	

Abbreviations: M—arithmetic mean, SD—standard deviation, *p*—probability value (Mann–Whitney U-test).

**Table 8:** Comparison of health practices before and during the COVID-19 pandemic in selected groups.

Sociodemographic Data		*n*	M ± SD Pre-Pandemic Health Practices	*p*	M ± SD during-Pandemic Health Practices	*p*
place of residence	urban	239	3.33 ± 0.67	0.600	3.47 ± 0.68	0.261
	rural	116	3.36 ± 0.55		3.56 ± 0.53	
number of children	primipara	116	3.41 ± 0.65	0.161	3.58 ± 0.71	0.120
	multipara	239	3.30 ± 0.62		3.46 ± 0.59	
COVID-19 vaccination	yes	186	3.31 ± 0.68	0.414	3.48 ± 0.66	0.626
	no	169	3.37 ± 0.58		3.53 ± 0.60	
anxiety and fear about the pandemic	yes	191	3.28 ± 0.64	0.080	3.47 ± 0.62	0.509
	no	164	3.41 ± 0.62		3.54 ± 0.65	

Abbreviations: M—arithmetic mean, SD—standard deviation, *p*—probability value (Mann–Whitney U-test).

## Data Availability

Data are available upon reasonable request.
